# TNF-α-induced up-regulation of pro-inflammatory cytokines is reduced by phosphatidylcholine in intestinal epithelial cells

**DOI:** 10.1186/1471-230X-9-53

**Published:** 2009-07-13

**Authors:** Irina Treede, Annika Braun, Petia Jeliaskova, Thomas Giese, Joachim Füllekrug, Gareth Griffiths, Wolfgang Stremmel, Robert Ehehalt

**Affiliations:** 1 Department of Gastroenterology, University of Heidelberg, INF 345, 69120 Heidelberg, Germany; 2 Institute of Immunology, University of Heidelberg, INF 305, 69120 Heidelberg, Germany; 3 Cell Biology Program, EMBL, Postfach 102209, 69117 Heidelberg, Germany

## Abstract

**Background:**

Phosphatidylcholine (PC) is a major lipid of the gastrointestinal mucus layer. We recently showed that mucus from patients suffering from ulcerative colitis has low levels of PC. Clinical studies reveal that the therapeutic addition of PC to the colonic mucus using slow release preparations is beneficial. The positive role of PC in this disease is still unclear; however, we have recently shown that PC has an intrinsic anti-inflammatory property. It could be demonstrated that the exogenous application of PC inhibits membrane-dependent actin assembly and TNF-α-induced nuclear NF-κB activation. We investigate here in more detail the hypothesis that the exogenous application of PC has anti-inflammatory properties.

**Methods:**

PC species with different fatty acid side chains were applied to differentiated and non-differentiated Caco-2 cells treated with TNF-α to induce a pro-inflammatory response. We analysed TNF-α-induced NF-κB-activation via the transient expression of a NF-κB-luciferase reporter system. Pro-inflammatory gene transcription was detected with the help of a quantitative real time (RT)-PCR analysis. We assessed the binding of TNF-α to its receptor by FACS and analysed lipid rafts by isolating detergent resistant membranes (DRMs).

**Results:**

The exogenous addition of all PC species tested significantly inhibited TNF-α-induced pro-inflammatory signalling. The expression levels of IL-8, ICAM-1, IP-10, MCP-1, TNF-α and MMP-1 were significantly reduced after PC pre-treatment for at least two hours. The effect was comparable to the inhibition of NF-kB by the NF-kB inhibitor SN 50 and was not due to a reduced binding of TNF-α to its receptor or a decreased surface expression of TNF-α receptors. PC was also effective when applied to the apical side of polarised Caco-2 cultures if cells were stimulated from the basolateral side. PC treatment changed the compartmentation of the TNF-α-receptors 1 and 2 to DRMs.

**Conclusion:**

PC induces a prolonged inhibition of TNF-α-induced pro-inflammatory signalling. This inhibition may be caused by a shift of the TNF-α receptors at the surface to lipid rafts. Our results may offer a potential molecular explanation for the positive role of PC seen in clinical studies for the treatment of ulcerative colitis.

## Background

Inflammatory bowel disease (IBD) is the result of a chronic intestinal inflammatory response. While the exact pathogenesis of IBD remains incompletely understood, it is likely that the initiation of the immune response is triggered by luminal factors [1]. The nature of these initiating agents is unclear, but both orally ingested nutrients and microbial agents have been implicated [2]. It is widely believed that an impaired barrier function, and in particular a defect of the mucus layer, leads to an increased exposure of the mucosal immune system to luminal antigens. In genetically susceptible individuals, this results in an inappropriate and unrestrained inflammatory response [3,4].

We have recently shown that both PC and lysophosphatidylcholine (LPC) but neither phosphatidylethanolamine (PE) nor sphingomyelin (SM) are decreased in the mucus of patients with ulcerative colitis (UC) and that the oral substitution of PC using slow release preparations is beneficial [5-7]. How luminal PC influences the clinical course of UC is not yet known, but two scenarios are possible. First, because of its hydrophobic property, PC coats the mucus layer, thereby preventing the contact of luminal bacteria to the mucosa [5,8,9]. With respect to this, luminal PC has been shown to synergize with conjugated primary bile salts in the binding of luminal endotoxin, which in turn leads to a suppressed inflammation beyond the mucosal surface [10]. On the other hand, we could recently demonstrate that PC per se has anti-inflammatory properties. PC has been shown to inhibit membrane-dependent actin assembly and TNF-α-induced MAPkinase and NF-κB activation [11]. In light of these results, we hypothesized that luminal PC might be integrated into the plasma membranes of enterocytes and in turn modulate the signalling state of the mucosa in the human intestine. This assumption is further substantiated by studies using an *in vitro *phagosome system [12,13] which show that exogenous PC is indeed involved in the networks which inhibit pro-inflammatory signalling in membranes.

IBD encompasses two chronic intestinal diseases, Crohn's disease (CD) and UC, which differ in their microscopic and macroscopic features although their symptoms are similar [14]. Ulcerative lesions in IBD are accompanied by a prominent infiltrate of inflammatory cells including T lymphocytes, macrophages, neutrophils, and mast and plasma cells [15]. Mechanisms involved in the recruiting and activating of these inflammatory cells are thought to encompass a complex interplay of inflammatory mediators. This is reflected by the elevation of various chemokines in the serum and mucosa of IBD patients (for review see [16]). Over 40 human chemokines are now acknowledged, each with its own specific pattern of cellular chemotaxis. The chemokine family is categorised into four groups depending on the spacing of their first two cysteine residues [17]. Cumulative studies demonstrate that all four types of chemokines are involved in the development of IBD [16]. The expression level of pro-inflammatory chemokines differed significantly between IBD patients and controls. Up-regulated chemokine expression in human biopsies correlated with increasing activity of the disease [16]. Pro-inflammatory cytokines such as TNF-α and interleukin 1β (IL-1β) up-regulate the transcription of chemokine genes and hence the synthesis of chemokines themselves through the activation of NF-κB [18,19]. As TNF-α has been shown to be an important player in the inflammatory process of IBD [20], we previously established a model cell system with human intestinal epithelial cells (Caco-2) which we stimulated with TNF-α to induce a pro-inflammatory response [11]. Using this system, we now analysed the effect of various PC species on the transcriptional levels of selected marker genes. After PC treatment, the TNF-α induced up-regulation was significantly reduced in a time- and dose-dependent manner depending on the fatty acid composition. PC was effective when applied to the apical side of polarized Caco-2 cells if they were stimulated from the basal side. We could show that the TNF-α effect was dependent on NF-κB activity and not due to inhibition of the binding of TNF-α to its receptor. PC treatment changed the compartmentation of TNF-α-R1 and TNF-α-R2 to lipid rafts, which is a possible mechanism of action.

## Methods

### Lipids and reagents

TNF-α was obtained from Promega (Mannheim, Germany) and dissolved in endotoxin-free water containing 1% BSA from Sigma (Deisenhofen, Germany). Aliquots of 10 μg/ml were stored at -70°C. The NF-κB inhibitor SN 50 was from Calbiochem (San Diego, USA). PC 18:2/18:2 (1,2-dilinoleoyl-glycero-3-PC), PC 16:0/16:0 (1,2-dipalmitoyl-glycero-3-PC), PC 16:0/18:2 (1-palmitoyl-2-linoleoyl-glycero-3-PC), LPC 16:0 (1-palmitoyl-glycero-3-PC), PE 16:1/16:1 (1,2-dioleyl-glycerol-3-PE) were from Sigma (Deisenhofen).

### Cells and bacteria

Caco-2w cells are a sub-clone of the human Caco-2BBe and were selected because of their well-differentiated phenotype. They were provided by J. R. Turner (University of Chicago). Cells were grown at 37°C and 5% CO_2 _in Dulbecco's modified Eagle's medium (DMEM) supplemented with 10% heat-inactivated fetal calf serum, 1% non-essential amino acid, and antibiotics (55 IU/mL penicillin and 55 μg/mL streptomycin). Vero cells (American Type Culture Collection; ATCC) were propagated in Dulbecco's Modified Eagle's Medium (DMEM) supplemented with 5% fetal bovine serum and antibiotics (55 IU/mL penicillin and 55 μg/mL streptomycin). For full polarization cells were grown for 14 d on 2.5 cm, 0.4 μm pore size Transwell polycarbonate filters (Costar, Cambridge, USA) as previously described [21].

### Gene expression analyses by RT-PCR quantification

Caco-2 cells were grown under standard conditions in 6-wells for 48 h to 90% confluence and washed in Dulbecco's modified Eagle's medium without serum. Then they were incubated with 10 ng/mL recombinant human TNF-α (RnD Systems, Wiesbaden-Nordenstadt, Germany) and different PC species for various times at 37°C. Approximately 1 × 10^6 ^cells were collected in 300 μl lysis buffer from the MagnaPure mRNA Isolation Kit I (RAS, Mannheim, Germany) and mRNA was isolated with the MagnaPure-LC device using the mRNA-I standard protocol. RNA was reverse transcribed using AMV-RT and oligo- (dT) as the primer (First Strand cDNA synthesis kit, RAS), according to the manufacturer's protocol, in a thermocycler. Primer sets specific for the sequences of the selected genes optimised for the LightCycler (RAS) were developed and provided by SEARCH-LC GmbH, Heidelberg http://www.search-lc.com. The PCR was performed with the LightCycler FastStart DNA Sybr GreenI kit (RAS) according to the protocol provided in the parameter specific kits. To control for the specificity of the amplification products, a melting curve analysis was performed. No amplification of unspecific products was observed. The copy number was calculated from a standard curve obtained by plotting known input concentrations of four different plasmids at log dilutions to the PCR cycle number at which the detected fluorescence intensity reaches a fixed value. This was done to reduce variations due to handling errors over several logarithmic dilution steps. To correct for differences in the content of mRNA, the calculated copy numbers were normalised according to the average expression of two housekeeping genes (Cyclophilin B and β-Actin). Values were thus given as input adjusted to the copy number per μl of cDNA.

### Transient transfection and reporter assays

Caco-2 cells were seeded in 12-wells, grown to a >95% confluency and transiently transfected with a NF-κB-dependent luciferase reporter plasmid according to the manufacturer's instructions (Stratagene, La Jolla, USA). Cells were then co-treated in medium with or without TNF-α (10 ng/ml) and with a 200 μM solution of different PCs for 4 h at 37°C 20 h after transfection. In pre-treatment experiments, cells were first incubated for 10 minutes with 200 μmol PC, washed, and afterwards stimulated with TNF-α (10 ng/ml) only. Luciferase activity was assayed using the Luciferase Assay Kit (Stratagene) according to the manufacturer's directions and detected with a Fluorostar Optima (BMG Labtech, Offenburg, Germany). Each transfection was performed in triplicate and repeated at least three times.

### Flow Cytometry

A 25 μl volume of washed cells (60% density, 2*10^5 ^cells), either pre-treated with a 200 μmol PC or left untreated, was incubated with 10 μl of biotinylated TNF-α (10 ng/mL) for 60 min at 4°C. Biotinylated soybean trypsin inhibitor was used as negative control. A 10 μl volume of avidin-FITC reagent was then added to each tube and incubated for an additional 30 min at 4°C in the dark. The cells were washed twice with 2 ml of 1× RDF1 buffer to remove unbound avidin-fluorescein and resuspended in 600 μl of 1× RDF1 buffer. The sample was then subjected to flow cytometric analysis by using 488 nm wavelength laser excitation (Beckman Coulter). As a test of specificity, TNF-α biotin was neutralized with an anti-TNF-α antibody and then added to the cells to block nonspecific and specific binding of TNF-α biotin.

### Preparation of DRMs

Detergent extraction with Triton X-100 was performed as described [22]. Cells were grown in 3.5 cm dishes, transfected with TNF-α-R1 and TNF-α-R2 (provided by J. R. Turner, University of Chicago) and 10–12 h later washed once with PBS and scraped on ice into 1.5 ml homogenisation buffer (250 mM Sucrose, 10 mM Hepes, 2 mM EDTA). After centrifugation (5 min at 2000 rpm), cell pellets were homogenised in a homogenisation buffer containing 20 μg/ml each of chymostatin, leupeptin, antipain and pepstatin A (Sigma) through a 26 G needle and centrifuged for 5 min at 3000 rpm. The resulting supernatant was subjected to extraction for 30 min at 4°C in 1% Triton X-100. The extracts were adjusted to 40% OptiPrep (Axis-Shield, Oslo, Norway) and overlaid in a TLS 55 centrifugation tube with 30% OptiPrep/TNE and TNE (25 mM Tris-HCl, pH 7.4, 150 mM NaCl, 2 mM EDTA old protocol/25 mM Tris-HCl, pH 10.8, 150 mM NaCl, 5 mM EDTA). The gradients were centrifuged at 400000 g in a Beckman SW41 rotor for 20 h at 4°C. Fractions were obtained and used for Western blotting as described [23]. Monoclonal antibodies against TNF-α receptor 1 (TNF-α-R1) were from Sigma and TNF-α receptor 2 (TNF-α-R2) from Alexis Biochemical (Lörrach, Germany); monoclonal antibodies against the Src-like kinase Yes were supplied from Transduction Laboratories (Lexington, K Y); and against Flotillin-1 came from BD Bioscience (Heidelberg, Germany).

### Statistical analysis

All values are reported as mean and standard deviation (SD) or standard error of the mean (SEM). For homogeneity of variance, ANOVA was applied. Significance levels between single groups (medium, +TNF-α, +TNF-α +phospholipid) were analysed using Tukey's post-hoc test. Probability values of p < 0.05 were set as a threshold for statistical significance.

## Results

### Different species of PC inhibit TNF-α-induced NF-κB activation in Caco-2 cells

Within our study, we tested the effect of several PC species (LPC 16:0, PC 18:2/18:2, PC 16:0/16:0, and PC 16:0/18:2) on the TNF-α-induced p65 promoter binding. First, Caco-2 cells were co-treated with TNF-α (10 ng) and PC. The promoter binding of p65 was detected by transient expression of a NF-κB luciferase reporter system and compared to the state of untreated cells. All PC species tested significantly inhibited the activation of NF-κB. As more unsaturated fatty acids the PC molecule had as more effective was the compound. The strongest effects were seen with LPC 16:0 (1-palmitoyl-glycero-3-PC) followed by PC 18:2/18:2 (1, 2-dilinoleoyl-glycero-3-PC). PC 16:0/16:0 was least effective. The effect was dose dependent for all PC species as previously shown for PC 18:2/18:2 [11] (see Additional file 1).

**Figure 1:**
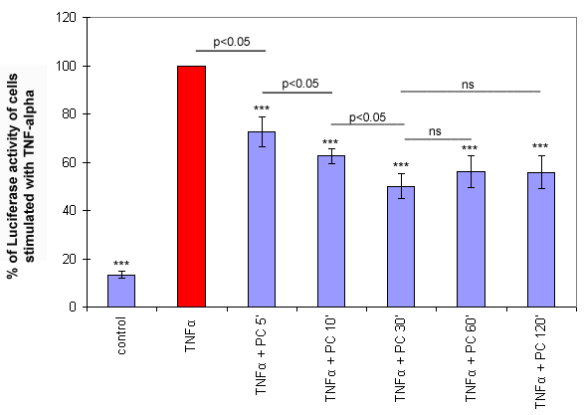
**Pre-incubation with a 200 μM solution of PC resulted in a time-dependent inhibition of TNF-α induced NF-κB activation**. NF-κB-activation was analysed via the transient expression of a NF-κB luciferase reporter system in Caco-2 cells. Cells were pre-treated with PC 16:0/16:0 (1, 2-dipalmitoyl-glycero-3-PC) for 5–120 min, washed and exposed for 4 h to 10 ng/ml TNF-α. The value was arbitrarily set to 100% in cells treated with TNF-α but not with lipid pre-treatment. Results presented here are representative for three others carried out independently. Data are expressed as mean and SD (n = 3). Asterisks assign statistically different values from Tukey's post hoc test compared to +TNF-α (*p < 0.05; **p < 0.01; ***p < 0.001).

Whenever a co-treatment experiment is performed, there is a risk that the applied substances could interfere with each other within the medium. Therefore, we also tested whether the pre-incubation of our cells with PC (pre-treatment) is effective. Cells were first incubated with the respective lipid, washed and subsequently stimulated with TNF-α. Interestingly, pre-treatment resulted in a time-dependent inhibition of the NF-κB activation. Within the first 30 min, NF-κB activation decreased with increasing time of pre-treatment. Pre-treatment for longer time periods did not show any additional effect (p > 0.05) (Figure 1). [3H]-PC uptake kinetics revealed that the internalisation of PC was almost linear within the first 5 min and thereafter reached a plateau that possible explains why no additional effect on NF-kB activation was seen with longer pre-treatment times (see Additional file 2[24]).

### Expression of pro-inflammatory genes is attenuated by PC

In previous publications, we have already shown that PC has an effect on pro-inflammatory gene transcription downstream of NF-κB [11]. In order to evaluate this effect in more detail, we analysed longer time periods and an extended set of genes. Caco-2 cells were stimulated with 10 ng/mL TNF-α for various times (30 to 240 min). Thereafter, mRNA was isolated to analyse the transcriptional levels by quantitative RT-PCR. Using this method, a significant up-regulation of IL-8, ICAM-1, IP-10, MCP-1, TNF-α and MMP-1 could be detected compared to the mRNA level of untreated cells (control) (p < 0.05) (see Additional file 3). Other genes (Cox-2, IL-10, IL-18, 4-1BB, ENA-78, TGFβ1) failed to be up-regulated by TNF-α (p > 0.05). After co-treatment of cells with PC 16:0/16:0 (1, 2-dipalmitoyl-glycero-3-PC) (+TNF-α +PC), the TNF-α effect was significantly reduced (p < 0.05). Similar effects were found for the treatment with LPC 16:0 (1-palmitoyl-glycero-3-PC). For evaluation of substrate specificity, we also tested the effect of PE 16:1/16:1 (1,2-dioleyl-glycerol-3-phosphatidylethanolamine (PE)). Co-treatment of cells with TNF-α and PE did not show any significant effect on the transcriptional level of ICAM-1, IP-10, MCP-1, TNF-α, MMP-1, Cox-2, IL-10, IL-18, 4-1BB, ENA-78 or TGFβ1 compared to TNF-α alone (p > 0.05).

Interestingly, some genes were up-regulated faster than others. This led us to classify the genes into two groups: 1) early up-regulated genes (IL-8, TNF-α), where a significant increase in transcript levels could be detected within 30 min; and 2) late up-regulated genes (ICAM-1, MCP-1, IP10, MMP-1) which responded later. PC pre-treatment reduced the transcriptional levels of both groups of genes significantly. The effect lasted for up to 120 min. Analysing later time points (240 min) did not show a significant difference anymore. The only exception was MMP-1, where a significant difference could also be detected after 240 min (see Additional file 3). This observation may be due to metabolic effects.

### The effect of PC on the expression levels of selected genes is comparable to the effect of the NF-κB inhibitor SN 50

It is known that the cytokine-induced expression of IL-8, MCP-1, COX-2 and other genes involved in inflammation is highly dependent on the transcription factor NF-κB [25-27]. An inhibitory effect of PC on the activation of the MAPkinases ERK and p38 have been previously described [11]. If we extend this by hypothesizing that PC inhibits TNF-α induced NF-κB activation and the subsequent pro-inflammatory gene transcription upstream of NF-κB, we could expect similar effects on gene transcription by inhibiting NF-κB in a direct way. Therefore we carried out experiments with the specific NF-κB-inhibitor SN 50. Pre-incubation of Caco-2 cells with SN 50 for 30 min before stimulation with TNF-α (10 ng/mL) resulted in a significantly reduced up-regulation of IL-8, ICAM-1, MCP-1 and IP-10. The inhibitory effects with SN 50 were as strong as the effects seen after pre-incubation with 200 μmol of either PC 16:0/16:0 (1, 2-dipalmitoyl-glycero-3-PC) (see Additional file 4) or LPC 16:0 (1-palmitoyl-glycero-3-PC) (data not shown). Therefore, the inhibitory effect of PC seems to be regulated within the TNF-α/NF-κB-pathway probably upstream of NF-κB.

### PC has no effect on the binding of TNF-α to its receptors

To exclude the possibility that PC might be acting by inhibiting the interaction of TNF-α to its receptors, we performed FACS analyses using biotin labeled TNF-α and avidin-FITC. The interaction of TNF-α with its receptor was readily and reproducibly observed in cells treated with either TNF-α-biotin alone or with cells pre-incubated for 1 h prior to the addition of TNF-α-biotin with 200 μmol of either PC 16:0/16:0 (1, 2-dipalmitoyl-glycero-3-PC) or PC 18:2/18:2 (1, 2-dilinoleoyl-glycero-3-PC). None of the tested PC species affected the interaction of TNF-α to its receptors or changed the density of TNF-α receptors at the cell surface (see Additional file 5).

### PC and LPC changes compartmentation of TNF-α-R1 and TNF-α-R2 to lipid rafts at the plasma membrane

**Figure 2:**
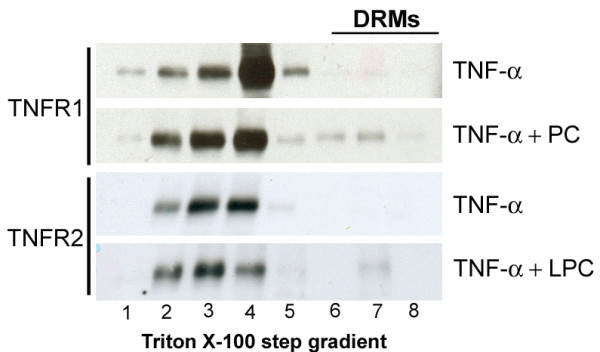
**This figure represents the effect of both LPC and PC on the association of TNF-α-R1 and TNF-α-R2 to DRMs**. COS cells were lysed in 1% Triton X-100 at 4°C 20 h after transient transfection of TNF-α-R1 and TNF-α-R2. After floatation in an OptiPrep step-gradient, TNF-α-R1 and TNF-α-R2 were found in two pools, in DRMs (lane 7–8) and in soluble membranes (lane 1–4). Pre-treatment with 200 μmol LPC or PC resulted in an increased DRM association.

We have previously shown that PC can inhibit the TNF-α-induced activation of the MAPkinases ERK and p38 [11]. If the effect of PC on TNF-α-induced gene transcription were indeed to take place upstream of NF-κB activation, it would possibly happen because of the lipophilic character of the substance within or at the plasma membrane. It has been previously shown that TNF-α receptors reside at the plasma membrane at least partially in lipid rafts and it has been suggested that compartmentation influences the signalling response [28]. As shown in Figure 2, PC does not change the concentration of TNF-α receptors at the cell surface. Therefore, we hypothesized that PC might not change the overall concentration but possibly the compartmentation of TNF-α receptors within the plasma membrane. DRMs were prepared in order to evaluate this hypothesis. Caco-2 cells were transfected with TNF-α-R1 and TNF-α-R2 and then extracted with Triton X-100 and subjected to OptiPrep step gradient centrifugation. TNF-α-R1 and TNF-α-R2 were found in two pools in cellular membranes, one within the DRMs and one outside of the DRMs. PC or LPC treatment shifted both receptors towards an increased DRM association (Figure 2). PC and LPC induced also an increased DRM association for the raft marker proteins Yes and Flotillin-1 (data not shown) that served as controls.

### PC inhibits the TNF-α-induced up-regulation of selected genes from the apical and basolateral sides of fully polarised Caco-2 cells

**Figure 3:**
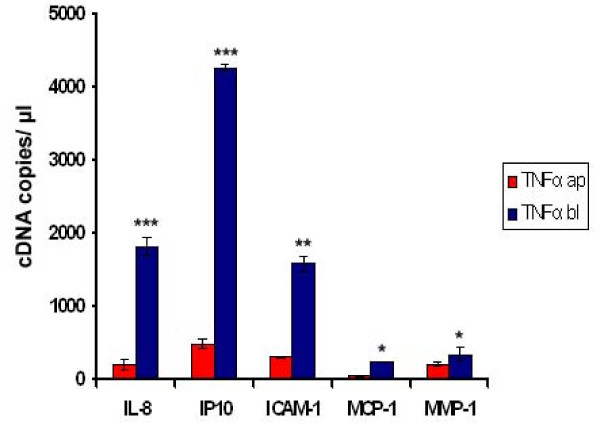
**This figure shows the effect of TNF-α from the apical and basal sides on gene transcription**. Caco-2 cells were grown in a 6-transwell system and stimulated with TNF-α (50 ng/mL) to induce an up-regulation of several selected genes involved in inflammation. Only basolateral stimulation with TNF-α (TNF-α bl) resulted in a significant up-regulation while apical stimulation with TNF-α (TNF-α ap) did not show any significant effect (p > 0.05). Data are expressed as mean and SD of n = 3 experiments. Asterisks assign statistically different values comparing apical and basolateral stimulation (*p < 0.05; **p < 0.01; ***p < 0.001).

**Figure 4:**
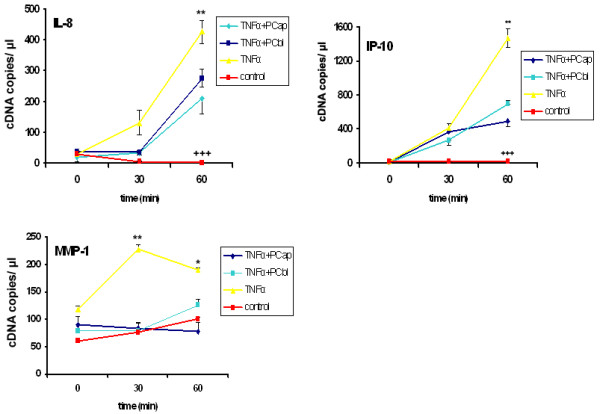
**PC inhibits pro-inflammatory gene transcription from both the apical and basolateral sides of polarised Caco-2 cells**. Polarised Caco-2 cells were grown in a 12-transwell system and stimulated with TNF-α (50 ng/mL) from the basolateral side to induce an up-regulation of several genes (IL-8, IP-10, and MMP-1) involved in inflammation (TNF-α). Both basolateral (+TNF-α +PC bl) and apical (+TNF-α +PC ap) treatments with 200 μmol of PC 16:0/16:0 (1, 2-dipalmitoyl-glycero-3-PC) resulted in a significant reduction in the TNF-α-induced up-regulation. Asterisks assign statistically different values from Tukey's post hoc test of TNF-α to all other values at each time point (*p < 0.05; **p < 0.01; ***p < 0.001); At no time point could a significant difference of +TNF-α +PC ap and +TNF-α +PC bl be detected; crosses indicate significant differences of control compared to both +TNF-α and +TNFα +PC at each time point (+ p < 0.05; ++ p < 0.01; +++ p < 0.001).

Based on the results with non-polarised cells, we also carried out analyses with fully polarised, filter grown Caco-2 cells. To establish this system, we tried out several conditions to stimulate the cells. It has been previously described that in fully polarised Caco-2 cells, the TNF-α-receptor is localised at the basolateral side and therefore not accessible for TNF-α applied to the apical surface [29,30]. We could confirm this finding by analysing the transcriptional level of selected genes after stimulating the cells with TNF-α from the apical and basolateral sides. An up-regulation could only be detected after stimulation from the basolateral side (Figure 3). Interestingly, PC and LPC inhibited TNF-α-induced gene transcription from both domains (Figure 4).

## Discussion

The data we have presented here further strengthens the evidence that the application of exogenous PC has anti-inflammatory effects. Firstly, we could confirm that different PC species can inhibit TNF-α-induced NF-κB activation. LPC functioned in the same way, whereas PE was not effective. The effect of PC was not merely transient but instead persisted for at least for 2 h. Consistent with previous results [11], we could demonstrate that the effect is dose dependent. Interestingly, PC 16:0/16:0 was less effective than species with unsaturated fatty acid side chains. As human mucus is made up of more than 90% PC species with one unsaturated fatty acid side chain (PC 16:0/18:1; PC 16:0/18:2; PC 18:0/18:1 and PC 18:0/18:2) [5], it is intriguing to speculate that this might represent an optimal mixture for controlling the inflammatory response. The hypothesis deduced from this is that mucus PC might be a source for membrane PC in enterocytes and thus influence membrane-dependent signalling of the mucosa cells.

Clinical and animal as well as in vitro data further support the anti-inflammatory effect of PC. We and others have previously shown that PC inhibits actin polymerisation on isolated phagosomes as well as in macrophages and Caco-2 cells [11,12]. In the latter model, it also inhibits activation of the MAPkinases ERK and p38, which are upstream of NF-κB [11]. Many animal studies support that PC is protective against pro-inflammatory conditions. With respect to this, it as been shown that it prevents mucosal injury induced by acids, NSAIDs or bile acids [9,31-39]. Parenteral administration of PC or LPC has been demonstrated to increase survival and improve inflammation in animal models of acute septic shock and endotoxemia [40-42]. Interestingly, there is a sudden overproduction of TNF-α in acute septic shock which triggers hypotension, circulatory collapse, and the widespread inflammation seen in this clinical condition [43]. Various species of LPC seem to protect against such a condition [42]. TNF-α is also thought to be an important agent in the colonic inflammation of patients with ulcerative colitis (UC). There is an increased density of TNF-α immunoreactive cells in the mucosa of UC patients with actual inflammation and there is an increased concentration of TNF-α in faeces, mucosa biopsies and serum of actively ill patients [44-46]. Most importantly, clinical studies have revealed that an anti-TNF-α strategy can be successful in treating those patients; therefore, this strategy is now integrated in daily clinical practice [47]. Two clinical studies on patients with ulcerative colitis have been performed which show a therapeutic benefit by adding PC in the form of slow release preparations [6,7]. In these studies, PC was applied in the form of soy lecithin with a considerable amount of PE. PE can be metabolised to PC via methylation and therefore serves as an additional source for PC within the cell. PE is also a known source for the endocannabinoid system, which influences inflammatory reactions as well [48]. However, our data show that exogenous PE has no significant effect on TNF-α-induced activation. This probably means that it is not the effective component of the preparation used in the clinical trials.

TNF-α exerts its functions through two distinct receptors, TNF-α-R1 (CD120a) and TNF-α-R2 (CD120b). After binding TNF-α, TNF-α-R1 recruits a death domain (DD)-containing adaptor molecule, namely the TNF-α-R1-associated death domain protein (TRADD). TRADD serves as a platform for recruiting additional mediators [49]. It binds the DD-containing Ser/Thr kinase receptor-interacting protein (RIP) and TNF-receptor-associated factor 2 (TRAF2). This TRADD-RIP-TRAF2 complex initiates the pathway leading to NF-kB activation [50-52]. How might exogenous PC function in this regulation?

1. It could be speculated that PC or LPC inhibits the association of TNF-α to its receptor. To exclude such an effect, we performed experiments in which the cells were treated with PC before TNF-α stimulation and quantified the binding of TNF-α to its receptor by FACS. FACS analysis could detect no quantitative differences between PC or LPC treated and non-treated cells. This makes a decreased receptor binding unlikely.

2. It has been previously suggested that TNF-α receptors, upon substrate binding, might be endocytosed and that the signalling response occurs in endosomes, as demonstrated in other receptors such as the β2-adrenergic receptor, the endothelin receptor and many other G-coupled protein receptors [53-56]. Thus it has been speculated that the integration of PC into cellular membranes might affect endocytosis and consequently signalling. However, we could show by FACS analysis that the surface expression of TNF-α receptors was unchanged after PC treatment. This makes an influence of PC on endocytosis also unlikely.

3. A third scenario seems more likely. Mammalian membranes are lipid bilayers consisting of 300–400 different lipid species that are ordered in vertical and lateral dimensions. This order is maintained with the help of a highly regulated concert of enzymes. Adding exogenous lipids would lead to a change in the lipid assembly of the membrane and therefore would influence membrane-dependent processes. We have previously speculated that PC might be present in a freely mobile form and a pool that is attached to proteins [11]. Proteins that bind selectively to PC have been described (e.g., START, C1 or C2 domain structures), of which a large number are involved in signalling [57,58]. Adding PC might shift the balance between the free and the bound fractions. Normally in cells the bulk of PC is in the outer leaflet of the membrane. It is therefore also possible that addition of PC might induce a higher concentration of PC on the inner leaflet. There it could interfere with selected signalling proteins such as MAP kinases or others involved in NF-κB activation. However, it has to be mentioned that the role of NF-κB is more complex in regulating the answer of the intestinal epithelium to luminal agents. NF-κB has been shown to regulate protective factors as well, including inducible beta defensins [59]. Epithelial-cell-specific inhibition of NF-kB through conditional ablation of NEMO (IKKgamma) led to an increased apoptosis of colonocytes, impaired expression of antimicrobial peptides and translocation of bacteria into the mucosa [60]. NF-κB therefore seems to be a regulator for the intestinal immune homoeostasis and the tuning of its activity by many mechanisms appears crucial in preventing inflammation [61]. The pool of membrane PC could be a part of this regulation.

4. Not only proteins but also membrane lipid compartments are necessary for TNF-α signalling to occur. It has been previously shown that TNF-α binding recruits TNF-α-R1 to lipid rafts [28]. Rafts are small platforms composed of sphingolipids and cholesterol in the outer exoplasmic leaflet and connected to phospholipids and cholesterol in the inner cytoplasmic leaflet of the lipid bilayer [62]. These assemblies are fluid but more ordered and tightly packed than the surrounding bilayer. The difference in packing is due to the saturation of the hydrocarbon chains in raft sphingolipids and phospholipids as compared with the unsaturated state of fatty acids of phospholipids in the surrounding bilayer [62]. Therefore it is possible that a change in the phospholipid concentration within the membrane has an impact on the lipid raft integrity. Discrepancies in the localisation of TNF-α-R1 to lipid rafts exist. Although in U937 and NIH-3T3 cells TNF-α-Rs were found mainly in lipid rafts and were shifted to detergent soluble parts upon TNF-α stimulation [63], TNF-α increased their lipid raft association in HeLa [64] and HT1080 fibrosarcoma cells [65]. Conflicting data also exist concerning a compartmentalised NF-kB activation. Whereas some authors claim that NF-kB activation by TNF-α occurs in lipid raft domains [28] other publications suggest a lipid-raft-independent activation [63]. However, our data showed that upon exposure of Caco-2 cells with PC or LPC, the lipid raft associations of both TNF-α-R1 and TNF-α-R2 are increased and both lipids were inhibitory in our setting. Therefore, we assume that TNF-α-induced NF-kB activation occurs outside lipid rafts.

To get closer to the scenario in the mucosa, polarised Caco-2 cells were analysed as well. Although polarised cells could only be stimulated by basolateral exposure to TNF-α, an inhibitory effect of PC could be observed from both the apical and basolateral sides. The cause of this phenomenon requires further investigation; however, a possible explanation may be that TNF-α signalling occurs after internalisation and that apical and basolateral vesicles mix with each other in the endosomal compartments [56]. Another possible explanation is that PC is integrated and rapidly exchanged between the apical and basolateral domains via the inner layer of the plasma membrane [66].

## Conclusion

In summary, our results further support the idea that exogenous PC and LPC have anti-inflammatory properties. Our findings suggest that PC (LPC) inhibits the signalling responses via NF-κB possibly due to changes in the plasma membrane. This is a potential mechanism explaining how PC can influence the course of ulcerative colitis and provides the molecular foundation for the benefit seen in our clinical studies.

## Abbreviations

DD: death domain; DRMs: detergent resistant membranes; IBD: inflammatory bowel disease; ICAM-1: intercellular adhesion molecule 1; IL-8, -1β: interleukin 8, 1β; IP-10: interferon inducible protein 10; LPC: lysophosphatidylcholine; MCP-1, -2, and -3: monocyte chemoattractant proteins 1, 2, and 3; NF-κB: nuclear factor-κB; ns: not significant; PC: phosphatidylcholine; PE: phosphatidylethanolamine; RIP: receptor-interacting protein; SD: standard deviation; SEM: standard error of the mean; SM: sphingomyelin; TNF-α: tumour necrosis factor α; TNF-α-R21, -R2: tumour necrosis factor α receptor 1, receptor 2; TRADD: TNF-α-R1-associated death domain protein; TRAF2: TNF-receptor-associated factor 2; UC: ulcerative colitis.

## Competing interests

The authors declare that they have no competing interests.

## Authors' contributions

RE precipitated in the design of the study and in the data analysis. He drafted the manuscript. AB, IT and PJ carried out the experiments. TG performed the RT-PCR analysis. JF, WS and GG were involved in the design of the study. All authors read and approved the final manuscript.

## Pre-publication history

The pre-publication history for this paper can be accessed here:

http://www.biomedcentral.com/1471-230X/9/53/prepub

## Supplementary Material

Additional file 1 **Co-treatment of Caco-2 cells with TNF-α and phospholipids**. Cells were grown in 3.5 cm dishes and the effects of different PC species on the inhibition of TNF-α-induced NF-κB-activation were analysed via the transient expression of a NF-κB-luciferase reporter system. (A) Cells were co-treated with 10 ng TNF-α and 200 μmol of the respective lipid. Luciferase activity was analysed 4 h after stimulation. Interestingly, PC 18:2/18:2 (1, 2-dilinoleoyl-glycero-3-PC), a PC species with two unsaturated side chains, was the most effective one compared to both PC 16:0/16:0 (1, 2-dipalmitoyl-glycero-3-PC) and PC 16:0/18:2 (1-palmitoyl-2-linoleoyl-glycero-3-PC) (p < 0.05). (B) Cells were co-treated with 10 ng/ml TNF-α and the indicated amounts of PC 16:0/16:0. PC inhibited NF-κB activation in a dose-dependent manner. The strongest effect was seen with 200 μmol PC which was significantly different from all other PC concentrations tested. The value was arbitrarily set to 100% in cells treated with TNF-α but not with phospholipids. Results presented here are representative for three others carried out independently. Data are shown as mean and SD (n = 3). Asterisks assign statistically different values from Tukey's post hoc test compared to control (*p < 0.05; **p < 0.01; ***p < 0.001); crosses indicate significant differences from +TNF-α after treatment with different phospholipids or phospholipid concentrations (+ p < 0.05; ++ p < 0.01; +++ p < 0.001). Click here for file

Additional file 2 **Uptake of [^3^H]-PC 16:0/16:0 into Caco-2 cells**. To investigate whether the time-dependent inhibition of NF-κB activation correlates to the amount of PC taken up into the cells, we analysed the uptake of [^3^H]-PC 16:0/16:0. Methods: Caco-2 cells were grown to 80% confluence in 5 cm^2 ^dishes and incubated for various times with different concentrations of 1-, 2-dipalmitoyl-3-phosphatidyl-[N-methyl-[^3^H]-choline ([^3^H]-PC, 81 Ci/mM) and 1-, 2-dipalmitoyl-glycero-3-phosphocholine (PC 16:0/16:0) at a ratio of 1:250. After washing, cells were incubated with NaOH (1 M) for 10 min. Both the cell lysates and the supernatants were analysed with counting solution in a scintillation counter (Beckman Coulter LS 6500) as done previously [11,24]. [^3^H]-methyl-choline]-L-dipalmitoyl-phosphatidylcholine ([^3^H]-PC) (50 Ci/mmol) was purchased from New England Nulcear, (Boston, MA, USA). Results: (A) The uptake reaches a plateau with increasing time. (B) Within the first 5 min of incubation with PC, the uptake was almost linear. An additional PC uptake was not detectable after 1 h. This uptake kinetic probably indicates that the inhibitory effect of exogenous PC correlates to the amount of PC incorporated into the cells. The plateau after 1 h likely explains why no additional effect on NF-κB activation was seen with longer pre-treatment times (Figure 1). Data are expressed as mean and SD of n = 5 experiments. Click here for file

Additional file 3 **Effect of PC 16:0/16:0 on up-regulation of selected pro-inflammatory genes after TNF-α stimulation**. Sub-confluent Caco-2 cells were stimulated with 10 ng/ml TNF-α to induce an up-regulation of MMP-1, IL-8, ICAM-1, MCP-1 and IP-10, which are known to be pro-inflammatory. (A) Co-treatment with 200 μmol of PC 16:0/16:0 (1, 2-dipalmitoyl-glycero-3-PC) resulted in a significant inhibition of the TNF-α-induced up-regulation. Up-regulation of selected genes after TNF-α stimulation might be classified into two groups: 1) early up-regulated genes (such as IL-8) and 2) late genes (ICAM-1, IP-10, MCP-1). The experiment depicted is representative of three others with the same results. Data are expressed as mean and SEM (n = 3). Asterisks assign statistically different values from Tukey's post hoc test (*p < 0.05; **p < 0.01; ***p < 0.001) comparing +TNF-α and +TNF-α +PC at each time point; crosses indicate significant differences of control compared to both +TNF-α and +TNF-α +PC at each time point (+ p < 0.05; ++ p < 0.01; +++ p < 0.001). Click here for file

Additional file 4 **Effect of SN 50 and PC on TNF-α-induced gene activation**. Sub-confluent Caco-2 cells were stimulated with TNF-α (10 ng/mL) to induce an up-regulation of several selected genes. Pre-treatment with the NF-κB inhibitor SN 50 (50 mg/mL) for 30 min resulted in a significant reduction of the TNF-α-induced up-regulation. This reduction was similar to that found in cells treated with PC, possibly indicating that both treatments influence the same pathway. Data are expressed as mean and SEM (n = 3). Asterisks assign statistically different values from Tukey's post hoc test of +TNF-α to all other values at each time point (*p < 0.05; **p < 0.01; ***p < 0.001); At no time point could a significant difference of +TNF-α +PC and +TNF-α +SN 50 be detected with the exception ICAM-1 at 120 min. Crosses indicate significant differences of control compared to +TNF-α, TNF-α +SN 50 and +TNF-α +PC at each time point (+ p < 0.05; ++ p < 0.01; +++ p < 0.001). Click here for file

Additional file 5 **Neither PC 16:0/16:0 nor PC 18:2/18:2 showed any effect on the binding of TNF-α to its cell surface receptors**. Vero cells were taken for FACS analyses because of their regular cell shape. They were either left untreated or were pre-treated for 1 h prior to the addition of TNF-α-biotin with a 200 μM preparation of PC 16:0/16:0 (1, 2-dipalmitoyl-glycero-3-PC) or PC 18:2/18:2 (1, 2-dilinoleoyl-glycero-3-PC). The PC-treated (TNF-α-biotin + PC 16:0/16:0 or TNF-α-biotin + PC 18:2/18:2) and untreated (TNF-α-biotin) cells were then collected for staining with biotinylated TNF-α and avidin-FITC. Soybean trypsin inhibitor that had been biotinylated in the same degree was used as a negative control. Neutralized TNF-α biotin with anti-TNF-α blocking antibody was used as a specificity control. Receptor binding activity was determined by flow cytometric analysis with a 488 nm wavelength laser excitation. Results shown here were representative for three other experiments carried out independently. Click here for file

## References

[B1] Podolsky DK Inflammatory bowel disease N Engl J Med 2002 347 6 417 429 10.1056/NEJMra020831 12167685

[B2] Hanauer SB Inflammatory bowel disease: epidemiology, pathogenesis, and therapeutic opportunities Inflamm Bowel Dis 2006 12 Suppl 1 S3 9 10.1097/01.MIB.0000195385.19268.68 16378007

[B3] Fiocchi C Inflammatory bowel disease: etiology and pathogenesis Gastroenterology 1998 115 1 182 205 10.1016/S0016-5085(98)70381-6 9649475

[B4] Wehkamp J Stange EF A new look at Crohn's disease: breakdown of the mucosal antibacterial defense Ann N Y Acad Sci 2006 1072 321 331 10.1196/annals.1326.030 17057212

[B5] Ehehalt R Wagenblast J Erben G Lehmann W Merle U Stremmel W Phosphatidylcholine and Lysophosphatidylcholine in Intestinal Mucus of Ulcerative Colitis Patients. A Quantitative Approach by nanoElectrospray-Tandem Mass Spectrometry Scand J Gastroenterol 2004 39 737 742 10.1080/00365520410006233 15513358

[B6] Stremmel W Merle U Zahn A Autschbach F Hinz U Ehehalt R Retarded release phosphatidylcholine benefits patients with chronic active ulcerative colitis Gut 2005 54 7 966 971 15951544 10.1136/gut.2004.052316 PMC1774598

[B7] Stremmel W Ehehalt R Autschbach F Karner M Phosphatidylcholine for steroid-refractory chronic ulcerative colitis: a randomized trial Ann Intern Med 2007 147 9 603 610 17975182 10.7326/0003-4819-147-9-200711060-00004

[B8] Bengmark S Jeppsson B Gastrointestinal surface protection and mucosa reconditioning JPEN J Parenter Enteral Nutr 1995 19 5 410 415 10.1177/0148607195019005410 8577022

[B9] Lichtenberger LM The hydrophobic barrier properties of gastrointestinal mucus Annu Rev Physiol 1995 57 565 583 10.1146/annurev.ph.57.030195.003025 7778878

[B10] Parlesak A Schaeckeler S Moser L Bode C Conjugated primary bile salts reduce permeability of endotoxin through intestinal epithelial cells and synergize with phosphatidylcholine in suppression of inflammatory cytokine production Crit Care Med 2007 35 10 2367 2374 10.1097/01.CCM.0000284586.84952.FB 17944028

[B11] Treede I Braun A Sparla R Kuhnel M Giese T Turner JR Anes E Kulaksiz H Fullekrug J Stremmel W Anti-inflammatory effects of phosphatidylcholine J Biol Chem 2007 282 37 27155 27164 17636253 10.1074/jbc.M704408200 PMC2693065

[B12] Anes E Kuhnel MP Bos E Moniz-Pereira J Habermann A Griffiths G Selected lipids activate phagosome actin assembly and maturation resulting in killing of pathogenic mycobacteria Nat Cell Biol 2003 5 9 793 802 10.1038/ncb1036 12942085

[B13] Miranda DT Batista VG Grando FC Paula FM Felicio CA Rubbo GF Fernandes LC Curi R Nishiyama A Soy lecithin supplementation alters macrophage phagocytosis and lymphocyte response to concanavalin A: a study in alloxan-induced diabetic rats Cell Biochem Funct 2008 26 8 859 865 10.1002/cbf.1517 18846580

[B14] Xavier RJ Podolsky DK Unravelling the pathogenesis of inflammatory bowel disease Nature 2007 448 7152 427 434 10.1038/nature06005 17653185

[B15] Strober W Fuss I Mannon P The fundamental basis of inflammatory bowel disease J Clin Invest 2007 117 3 514 521 17332878 10.1172/JCI30587 PMC1804356

[B16] Zhong W Kolls JK Chen H McAllister F Oliver PD Zhang Z Chemokines orchestrate leukocyte trafficking in inflammatory bowel disease Front Biosci 2008 13 1654 1664 10.2741/2789 17981657

[B17] Laing KJ Secombes CJ Chemokines Dev Comp Immunol 2004 28 5 443 460 10.1016/j.dci.2003.09.006 15062643

[B18] Elewaut D DiDonato JA Kim JM Truong F Eckmann L Kagnoff MF NF-kappa B is a central regulator of the intestinal epithelial cell innate immune response induced by infection with enteroinvasive bacteria J Immunol 1999 163 3 1457 1466 10415047

[B19] Jung HC Eckmann L Yang SK Panja A Fierer J Morzycka-Wroblewska E Kagnoff MF A distinct array of proinflammatory cytokines is expressed in human colon epithelial cells in response to bacterial invasion J Clin Invest 1995 95 1 55 65 7814646 10.1172/JCI117676 PMC295369

[B20] van Assche G Vermeire S Rutgeerts P Emerging biological treatments in inflammatory bowel diseases Minerva Gastroenterol Dietol 2007 53 3 249 255 17912187

[B21] Ehehalt R Krautter M Zorn M Sparla R Fullekrug J Kulaksiz H Stremmel W Increased basolateral sorting of carcinoembryonic antigen in a polarized colon carcinoma cell line after cholesterol depletion-Implications for treatment of inflammatory bowel disease World J Gastroenterol 2008 14 10 1528 1533 18330942 10.3748/wjg.14.1528 PMC2693746

[B22] Ehehalt R Keller P Haass C Thiele C Simons K Amyloidogenic processing of the Alzheimer beta-amyloid precursor protein depends on lipid rafts J Cell Biol 2003 160 1 113 123 12515826 10.1083/jcb.200207113 PMC2172747

[B23] Pohl J Ring A Korkmaz U Ehehalt R Stremmel W FAT/CD36 Mediated Long-Chain Fatty Acid Uptake in Adipocytes Requires Plasma Membrane Rafts Mol Biol Cell 2004 16 24 31 15496455 10.1091/mbc.E04-07-0616 PMC539148

[B24] Ehehalt R Jochims C Lehmann WD Erben G Staffer S Reininger C Stremmel W Evidence of luminal phosphatidylcholine secretion in rat ileum Biochim Biophys Acta 2004 1682 1–3 63 71 15158757 10.1016/j.bbalip.2004.01.009

[B25] Ueda A Ishigatsubo Y Okubo T Yoshimura T Transcriptional regulation of the human monocyte chemoattractant protein-1 gene. Cooperation of two NF-kappaB sites and NF-kappaB/Rel subunit specificity J Biol Chem 1997 272 49 31092 31099 10.1074/jbc.272.49.31092 9388261

[B26] Matsusaka T Fujikawa K Nishio Y Mukaida N Matsushima K Kishimoto T Akira S Transcription factors NF-IL6 and NF-kappa B synergistically activate transcription of the inflammatory cytokines, interleukin 6 and interleukin 8 Proc Natl Acad Sci USA 1993 90 21 10193 10197 8234276 10.1073/pnas.90.21.10193 PMC47740

[B27] Moriuchi H Moriuchi M Fauci AS Nuclear factor-kappa B potently up-regulates the promoter activity of RANTES, a chemokine that blocks HIV infection J Immunol 1997 158 7 3483 3491 9120310

[B28] Legler DF Micheau O Doucey MA Tschopp J Bron C Recruitment of TNF receptor 1 to lipid rafts is essential for TNFalpha-mediated NF-kappaB activation Immunity 2003 18 5 655 664 10.1016/S1074-7613(03)00092-X 12753742

[B29] Fish SM Proujansky R Reenstra WW Synergistic effects of interferon gamma and tumour necrosis factor alpha on T84 cell function Gut 1999 45 2 191 198 10403730 10.1136/gut.45.2.191 PMC1727614

[B30] Vallee S Laforest S Fouchier F Montero MP Penel C Champion S Cytokine-induced upregulation of NF-kappaB, IL-8, and ICAM-1 is dependent on colonic cell polarity: implication for PKCdelta Exp Cell Res 2004 297 1 165 185 10.1016/j.yexcr.2004.03.007 15194434

[B31] Dial EJ Lichtenberger LM A role for milk phospholipids in protection against gastric acid. Studies in adult and suckling rats Gastroenterology 1984 87 2 379 385 6610595

[B32] Dunjic BS Axelson J Ar'Rajab A Larsson K Bengmark S Gastroprotective capability of exogenous phosphatidylcholine in experimentally induced chronic gastric ulcers in rats Scand J Gastroenterol 1993 28 1 89 94 10.3109/00365529309096051 8430278

[B33] el-Hariri LM Marriott C Martin GP The mitigating effects of phosphatidylcholines on bile salt- and lysophosphatidylcholine-induced membrane damage J Pharm Pharmacol 1992 44 8 651 654 1359088 10.1111/j.2042-7158.1992.tb05487.x

[B34] Fabia R Ar'Rajab A Willen R Andersson R Ahren B Larsson K Bengmark S Effects of phosphatidylcholine and phosphatidylinositol on acetic-acid-induced colitis in the rat Digestion 1992 53 1–2 35 44 10.1159/000200969 1289171

[B35] Hills BA Kirwood CA Surfactant approach to the gastric mucosal barrier: protection of rats by banana even when acidified Gastroenterology 1989 97 2 294 303 2744353 10.1016/0016-5085(89)90064-4

[B36] Kiviluoto T Paimela H Mustonen H Kivilaakso E Exogenous surface-active phospholipid protects Necturus gastric mucosa against luminal acid and barrier-breaking agents Gastroenterology 1991 100 1 38 46 1983849 10.1016/0016-5085(91)90580-e

[B37] Lugea A Mourelle M Guarner F Domingo A Salas A Malagelada JR Phosphatidylcholines as mediators of adaptive cytoprotection of the rat duodenum Gastroenterology 1994 107 3 720 727 8076757 10.1016/0016-5085(94)90119-8

[B38] Mourelle M Guarner F Malagelada JR Polyunsaturated phosphatidylcholine prevents stricture formation in a rat model of colitis Gastroenterology 1996 110 4 1093 1097 10.1053/gast.1996.v110.pm8612998 8612998

[B39] Nervi F Significance of biliary phospholipids for maintenance of the gastrointestinal mucosal barrier and hepatocellular integrity Gastroenterology 2000 118 6 1265 1267 10.1016/S0016-5085(00)70380-5 10833502

[B40] Drobnik W Liebisch G Audebert FX Frohlich D Gluck T Vogel P Rothe G Schmitz G Plasma ceramide and lysophosphatidylcholine inversely correlate with mortality in sepsis patients J Lipid Res 2003 44 4 754 761 10.1194/jlr.M200401-JLR200 12562829

[B41] Ilcol YO Yilmaz Z Ulus IH Endotoxin alters serum-free choline and phospholipid-bound choline concentrations, and choline administration attenuates endotoxin-induced organ injury in dogs Shock 2005 24 3 288 293 10.1097/01.shk.0000174018.02688.4b 16135970

[B42] Yan JJ Jung JS Lee JE Lee J Huh SO Kim HS Jung KC Cho JY Nam JS Suh HW Therapeutic effects of lysophosphatidylcholine in experimental sepsis Nat Med 2004 10 2 161 167 10.1038/nm989 14716308

[B43] Tracey KJ Fong Y Hesse DG Manogue KR Lee AT Kuo GC Lowry SF Cerami A Anti-cachectin/TNF monoclonal antibodies prevent septic shock during lethal bacteraemia Nature 1987 330 6149 662 664 10.1038/330662a0 3317066

[B44] Komatsu M Kobayashi D Saito K Furuya D Yagihashi A Araake H Tsuji N Sakamaki S Niitsu Y Watanabe N Tumor necrosis factor-alpha in serum of patients with inflammatory bowel disease as measured by a highly sensitive immuno-PCR Clin Chem 2001 47 7 1297 1301 11427462

[B45] Casellas F Papo M Guarner F Antolin M Armengol JR Malagelada JR Intraluminal colonic release of immunoreactive tumour necrosis factor in chronic ulcerative colitis Clin Sci (Lond) 1994 87 4 453 458 7834999 10.1042/cs0870453

[B46] Murch SH Braegger CP Walker-Smith JA MacDonald TT Location of tumour necrosis factor alpha by immunohistochemistry in chronic inflammatory bowel disease Gut 1993 34 12 1705 1709 8031350 10.1136/gut.34.12.1705 PMC1374467

[B47] Velayos FS Sandborn WJ Positioning biologic therapy for Crohn's disease and ulcerative colitis Curr Gastroenterol Rep 2007 9 6 521 527 10.1007/s11894-007-0069-1 18377806

[B48] Ashton JC Cannabinoids for the treatment of inflammation Curr Opin Investig Drugs 2007 8 5 373 384 17520866

[B49] Hsu H Xiong J Goeddel DV The TNF receptor 1-associated protein TRADD signals cell death and NF-kappa B activation Cell 1995 81 4 495 504 10.1016/0092-8674(95)90070-5 7758105

[B50] Hsu H Shu HB Pan MG Goeddel DV TRADD-TRAF2 and TRADD-FADD interactions define two distinct TNF receptor 1 signal transduction pathways Cell 1996 84 2 299 308 10.1016/S0092-8674(00)80984-8 8565075

[B51] Devin A Cook A Lin Y Rodriguez Y Kelliher M Liu Z The distinct roles of TRAF2 and RIP in IKK activation by TNF-R1: TRAF2 recruits IKK to TNF-R1 while RIP mediates IKK activation Immunity 2000 12 4 419 429 10.1016/S1074-7613(00)80194-6 10795740

[B52] Kelliher MA Grimm S Ishida Y Kuo F Stanger BZ Leder P The death domain kinase RIP mediates the TNF-induced NF-kappaB signal Immunity 1998 8 3 297 303 10.1016/S1074-7613(00)80535-X 9529147

[B53] Cao TT Mays RW von Zastrow M Regulated endocytosis of G-protein-coupled receptors by a biochemically and functionally distinct subpopulation of clathrin-coated pits J Biol Chem 1998 273 38 24592 24602 10.1074/jbc.273.38.24592 9733754

[B54] Bremnes T Paasche JD Mehlum A Sandberg C Bremnes B Attramadal H Regulation and intracellular trafficking pathways of the endothelin receptors J Biol Chem 2000 275 23 17596 17604 10.1074/jbc.M000142200 10747877

[B55] Sorkin A Von Zastrow M Signal transduction and endocytosis: close encounters of many kinds Nat Rev Mol Cell Biol 2002 3 8 600 614 10.1038/nrm883 12154371

[B56] Escobar GA McIntyre RC Jr Moore EE Gamboni-Robertson F Banerjee A Clathrin heavy chain is required for TNF-induced inflammatory signaling Surgery 2006 140 2 268 272 10.1016/j.surg.2006.03.008 16904979

[B57] Alpy F Tomasetto C Give lipids a START: the StAR-related lipid transfer (START) domain in mammals J Cell Sci 2005 118 Pt 13 2791 2801 10.1242/jcs.02485 15976441

[B58] Hurley JH Tsujishita Y Pearson MA Floundering about at cell membranes: a structural view of phospholipid signaling Curr Opin Struct Biol 2000 10 6 737 743 10.1016/S0959-440X(00)00144-5 11114512

[B59] Wehkamp J Harder J Wehkamp K Wehkamp-von Meissner B Schlee M Enders C Sonnenborn U Nuding S Bengmark S Fellermann K NF-kappaB- and AP-1-mediated induction of human beta defensin-2 in intestinal epithelial cells by Escherichia coli Nissle 1917: a novel effect of a probiotic bacterium Infect Immun 2004 72 10 5750 5758 15385474 10.1128/IAI.72.10.5750-5758.2004 PMC517557

[B60] Nenci A Becker C Wullaert A Gareus R van Loo G Danese S Huth M Nikolaev A Neufert C Madison B Epithelial NEMO links innate immunity to chronic intestinal inflammation Nature 2007 446 7135 557 561 10.1038/nature05698 17361131

[B61] Atreya I Atreya R Neurath MF NF-kappaB in inflammatory bowel disease J Intern Med 2008 263 6 591 596 10.1111/j.1365-2796.2008.01953.x 18479258

[B62] Simons K Ehehalt R Cholesterol, lipid rafts, and disease J Clin Invest 2002 110 5 597 603 12208858 10.1172/JCI16390 PMC151114

[B63] Ko YG Lee JS Kang YS Ahn JH Seo JS TNF-alpha-mediated apoptosis is initiated in caveolae-like domains J Immunol 1999 162 12 7217 7223 10358168

[B64] Cottin V Doan JE Riches DW Restricted localization of the TNF receptor CD120a to lipid rafts: a novel role for the death domain J Immunol 2002 168 8 4095 4102 11937569 10.4049/jimmunol.168.8.4095

[B65] Veldman RJ Maestre N Aduib OM Medin JA Salvayre R Levade T A neutral sphingomyelinase resides in sphingolipid-enriched microdomains and is inhibited by the caveolin-scaffolding domain: potential implications in tumour necrosis factor signalling Biochem J 2001 355 Pt 3 859 868 11311151 10.1042/bj3550859 PMC1221804

[B66] Simons K van Meer G Lipid sorting in epithelial cells Biochemistry 1988 27 17 6197 6202 10.1021/bi00417a001 3064805

