# Supplementary figures and images for: TNF-α-induced up-regulation of pro-inflammatory cytokines is reduced by phosphatidylcholine in intestinal epithelial cells
Source: BMC Gastroenterol. 2009 Jul 13;9:53. doi: 10.1186/1471-230X-9-53 (PMC2714528; doi:10.1186/1471-230X-9-53)

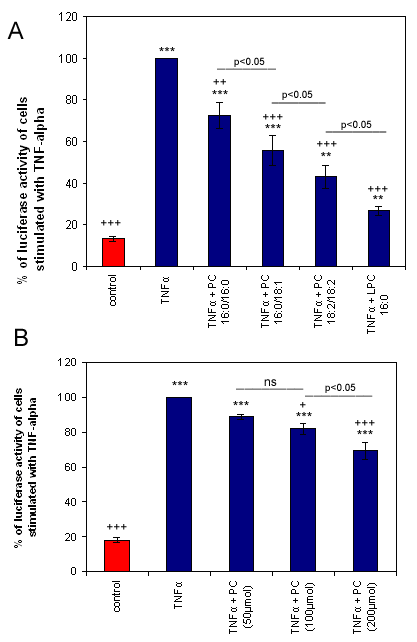

Supplement: Additional file 1 — Co-treatment of Caco-2 cells with TNF-α and phospholipids. Cells were grown in 3.5 cm dishes and the effects of different PC species on the inhibition of TNF-α-induced NF-κB-activation were analysed via the transient expression of a NF-κB-luciferase reporter system. (A) Cells were co-treated with 10 ng TNF-α and 200 μmol of the respective lipid. Luciferase activity was analysed 4 h after stimulation. Interestingly, PC 18:2/18:2 (1, 2-dilinoleoyl-glycero-3-PC), a PC species with two unsaturated side chains, was the most effective one compared to both PC 16:0/16:0 (1, 2-dipalmitoyl-glycero-3-PC) and PC 16:0/18:2 (1-palmitoyl-2-linoleoyl-glycero-3-PC) (p < 0.05). (B) Cells were co-treated with 10 ng/ml TNF-α and the indicated amounts of PC 16:0/16:0. PC inhibited NF-κB activation in a dose-dependent manner. The strongest effect was seen with 200 μmol PC which was significantly different from all other PC concentrations tested. The value was arbitrarily set to 100% in cells treated with TNF-α but not with phospholipids. Results presented here are representative for three others carried out independently. Data are shown as mean and SD (n = 3). Asterisks assign statistically different values from Tukey's post hoc test compared to control (*p < 0.05; **p < 0.01; ***p < 0.001); crosses indicate significant differences from +TNF-α after treatment with different phospholipids or phospholipid concentrations (+ p < 0.05; ++ p < 0.01; +++ p < 0.001). [file 1471-230X-9-53-S1.tiff]

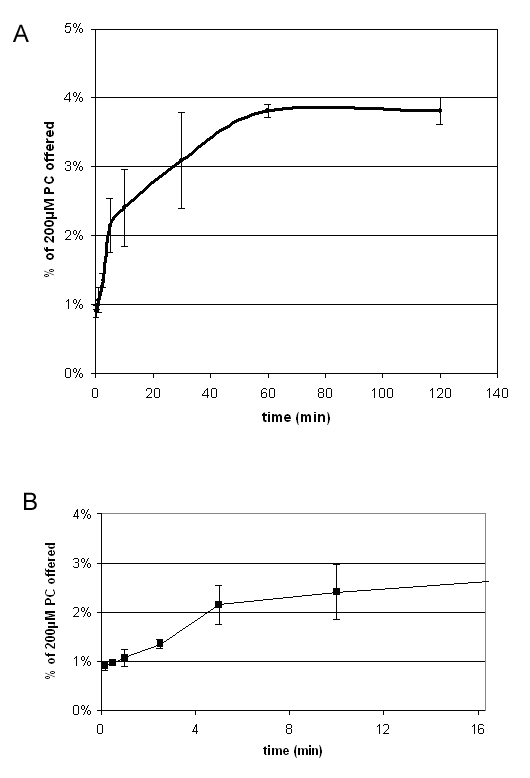

Supplement: Additional file 2 — Uptake of [3H]-PC 16:0/16:0 into Caco-2 cells. To investigate whether the time-dependent inhibition of NF-κB activation correlates to the amount of PC taken up into the cells, we analysed the uptake of [3H]-PC 16:0/16:0. Methods: Caco-2 cells were grown to 80% confluence in 5 cm2 dishes and incubated for various times with different concentrations of 1-, 2-dipalmitoyl-3-phosphatidyl-[N-methyl-[3H]-choline ([3H]-PC, 81 Ci/mM) and 1-, 2-dipalmitoyl-glycero-3-phosphocholine (PC 16:0/16:0) at a ratio of 1:250. After washing, cells were incubated with NaOH (1 M) for 10 min. Both the cell lysates and the supernatants were analysed with counting solution in a scintillation counter (Beckman Coulter LS 6500) as done previously [11,24]. [3H]-methyl-choline]-L-dipalmitoyl-phosphatidylcholine ([3H]-PC) (50 Ci/mmol) was purchased from New England Nulcear, (Boston, MA, USA). Results: (A) The uptake reaches a plateau with increasing time. (B) Within the first 5 min of incubation with PC, the uptake was almost linear. An additional PC uptake was not detectable after 1 h. This uptake kinetic probably indicates that the inhibitory effect of exogenous PC correlates to the amount of PC incorporated into the cells. The plateau after 1 h likely explains why no additional effect on NF-κB activation was seen with longer pre-treatment times (Figure 1). Data are expressed as mean and SD of n = 5 experiments. [file 1471-230X-9-53-S2.tiff]

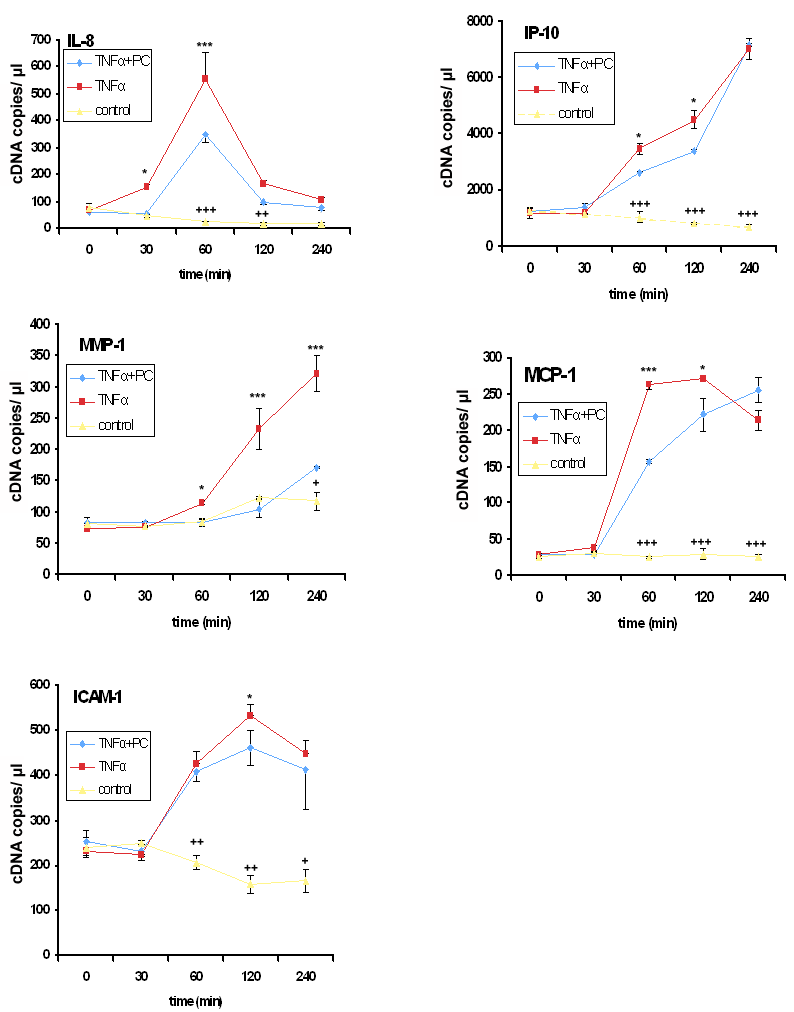

Supplement: Additional file 3 — Effect of PC 16:0/16:0 on up-regulation of selected pro-inflammatory genes after TNF-α stimulation. Sub-confluent Caco-2 cells were stimulated with 10 ng/ml TNF-α to induce an up-regulation of MMP-1, IL-8, ICAM-1, MCP-1 and IP-10, which are known to be pro-inflammatory. (A) Co-treatment with 200 μmol of PC 16:0/16:0 (1, 2-dipalmitoyl-glycero-3-PC) resulted in a significant inhibition of the TNF-α-induced up-regulation. Up-regulation of selected genes after TNF-α stimulation might be classified into two groups: 1) early up-regulated genes (such as IL-8) and 2) late genes (ICAM-1, IP-10, MCP-1). The experiment depicted is representative of three others with the same results. Data are expressed as mean and SEM (n = 3). Asterisks assign statistically different values from Tukey's post hoc test (*p < 0.05; **p < 0.01; ***p < 0.001) comparing +TNF-α and +TNF-α +PC at each time point; crosses indicate significant differences of control compared to both +TNF-α and +TNF-α +PC at each time point (+ p < 0.05; ++ p < 0.01; +++ p < 0.001). [file 1471-230X-9-53-S3.tiff]

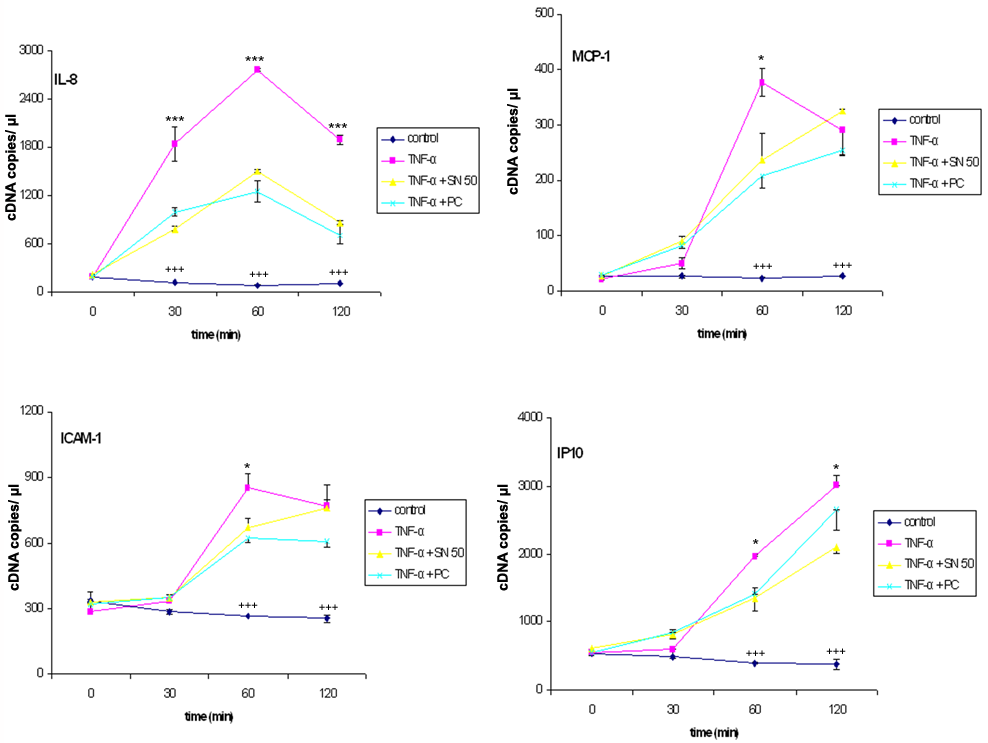

Supplement: Additional file 4 — Effect of SN 50 and PC on TNF-α-induced gene activation. Sub-confluent Caco-2 cells were stimulated with TNF-α (10 ng/mL) to induce an up-regulation of several selected genes. Pre-treatment with the NF-κB inhibitor SN 50 (50 mg/mL) for 30 min resulted in a significant reduction of the TNF-α-induced up-regulation. This reduction was similar to that found in cells treated with PC, possibly indicating that both treatments influence the same pathway. Data are expressed as mean and SEM (n = 3). Asterisks assign statistically different values from Tukey's post hoc test of +TNF-α to all other values at each time point (*p < 0.05; **p < 0.01; ***p < 0.001); At no time point could a significant difference of +TNF-α +PC and +TNF-α +SN 50 be detected with the exception ICAM-1 at 120 min. Crosses indicate significant differences of control compared to +TNF-α, TNF-α +SN 50 and +TNF-α +PC at each time point (+ p < 0.05; ++ p < 0.01; +++ p < 0.001). [file 1471-230X-9-53-S4.tiff]

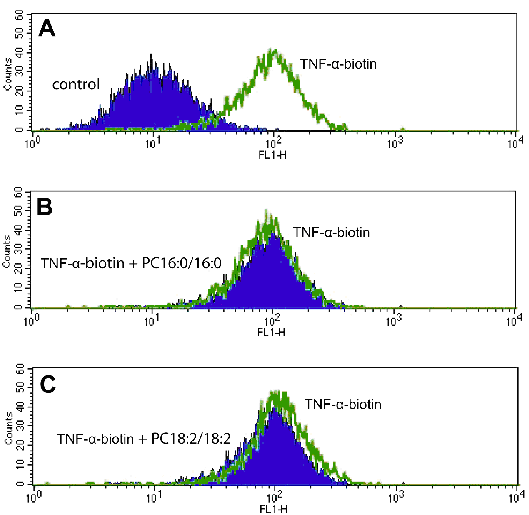

Supplement: Additional file 5 — Neither PC 16:0/16:0 nor PC 18:2/18:2 showed any effect on the binding of TNF-α to its cell surface receptors. Vero cells were taken for FACS analyses because of their regular cell shape. They were either left untreated or were pre-treated for 1 h prior to the addition of TNF-α-biotin with a 200 μM preparation of PC 16:0/16:0 (1, 2-dipalmitoyl-glycero-3-PC) or PC 18:2/18:2 (1, 2-dilinoleoyl-glycero-3-PC). The PC-treated (TNF-α-biotin + PC 16:0/16:0 or TNF-α-biotin + PC 18:2/18:2) and untreated (TNF-α-biotin) cells were then collected for staining with biotinylated TNF-α and avidin-FITC. Soybean trypsin inhibitor that had been biotinylated in the same degree was used as a negative control. Neutralized TNF-α biotin with anti-TNF-α blocking antibody was used as a specificity control. Receptor binding activity was determined by flow cytometric analysis with a 488 nm wavelength laser excitation. Results shown here were representative for three other experiments carried out independently. [file 1471-230X-9-53-S5.tiff]
